# A General System of Toxicology, or a Treatise on Poisons Drawn from the Mineral, Vegetable, and Animal Kingdoms, &c.

**Published:** 1816-07

**Authors:** 


*Traité des Poisons tirés des Regnes Minéral, Végétal, et Animal, ou Toxicologie Générale, &c.* Par M. P. Orfila, D.M. &c.

---

*A General System of Toxicology, or a Treatise on Poisons drawn from the Mineral, Vegetable, and Animal Kingdoms, &c.* By M. P. Orfila, M. D. &c. *Translated from the French.* Vol. I. Parts I and II.

( Continued from Vol. i. page 515. )

Our present sketch will conclude the first volume. We are still on the *corrosive class* of poisons.

*Article* 11. *Species* 11. *Pure and carbonated Alkalis.* Var. 1. *Potash*; 2. *Soda*; 3. *Ammonia*, in their *caustic* and *carbonated* states. The *physical characters* of all these substances, we think it needless to describe. *Chemical:* The three *pure* alkalis possess, in common, the properties of turning syrup of violets green, and restoring the colour of litmus reddened by an acid; often rendering *red wine* green; exerting no sensible action on *sugared water, tea, albumen, gelatine, milk*, or *bile*, and of not coagulating *fluid blood*. Potash and ammonia precipitate deuto-muriate of platina of an orange-yellow; soda does *not*. The sulphates of the two former alkalis throw down chrystals of alum from concentrated solution of the acid sulphate of alumina; sulphate of soda exhibits *not* this phænomenon. The solution of potash is *not* rendered turbid by the addition of carbonic acid gas; and hence may be distinguished from barytes, strontian, and lime: it forms a blackish-brown precipitate with nitrate of silver. Ammonia, on the other hand, does *not* disturb the nitrate of silver; and may be clearly recognized from potash and soda by adding it, in excess, to an aqueous solution of sulphate of magnesia: here, the magnesia is only partially precipitated; * whereas the whole is thrown down by the other two alkalis. Of the *carbonates*, little needs be said; they exert the same operation as their respective bases upon the alimentary and animal fluids just enumerated. *Physiological action:* All these substances, when injected into the veins, destroy life by coagulating the blood. Introduced into the stomach, they induce gastritis frequently terminating in gangrene and perforation. Ammonia also exerts an action on the nervous system, particularly on the vertebral column. *Symptoms:* Acrid, urinous, caustic taste; heat in the throat; vomiting of matter often bloody, alkaline, rendering green the syrup of violets, and commonly effervescing with acids; copious diarrhœa; severe gastric pains; violent colic, convulsions, and disorder of the intellect. Death speedily follows if the dose be large. Pure ammonia is said to have destroyed life in a few minutes, after having burned the lips, tongue, and palate, and determined intestinal and nasal hæmorrhage, and hectic fever. *Organic lesions:* those which mark the operation of the other corrosives;

---

* The liquid, in this case, contains a triple compound of ammonia, magnesia, and sulphuric acid; and, when filtered, deposits, on the addition of potash, a fresh quantity of magnesia.

but M. Orfila is "induced to believe" that potash (and we presume soda also) more frequently produces perforation of the stomach, than any other poison of the corrosive class. A retrospect of the facts developed in the chemical history of these substances, will suffice to indicate the *conduct which the practitioner should pursue* in detecting the precise nature of an alkaline poison in any given case. We shall merely remind him that potash may be discriminated from soda by its effect upon deuto-muriate of platina; from ammonia, by absence of smell, and its operation on sulphate of magnesia; from lime, strontian, and barytes, by its insensibility to carbonic acid: it may, also, be always procured in a solid state from liquids by evaporation. The presence of ammonia in a fluid may be ascertained by distilling it in a retort, to which a receiver, containing a little water, has been adapted. The ammonia, thus volatilized, will saturate the water: and some pieces of litmus paper, reddened by an acid, and fixed to the interior of the receiver, will, by restoration of their colour, announce the presence of ammoniacal gas. *Treatment. Vinegar diluted with water and largely given,* will both neutralize the poison, and provoke vomiting: Water, cold or tepid, and mucilaginous fluids, should be copiously thrown in. Inflammation of the internal organs is to be obviated or subdued by the usual means. When *ammonia* has been swallowed, the most prompt and energetic measures will be necessary, from the rapidity of its operation upon the nervous system.

*Article* 12. *Species* 12. *Caustic alkaline earths.* Var. 1. *Barytes;* 2. *Lime. Muriate and carbonate of barytes* are also included; but we must chiefly restrict our observations to the *simple earth. Barytes. Phys. and Chem. characters:* Solid; colour greenish-grey; taste acrid and caustic; when calcined, it absorbs water, gives out heat, swells, and falls into a whitish powder, soluble in distilled water at somewhat above the common temperature. The solution, limpid, transparent, colourless, renders syrup of violets green; tumeric paper red; and restores the colour of litmus reddened by an acid: precipitated white by carbonic acid gas; carbonic-acid-water,* alkaline subcarbonates; sulphuric acid and the soluble sulphates: renders *wine* slightly turbid; precipitates *human bile* of a greenish-yellow: does not disturb *tea,* but gives it the

* Water strongly impregnated with this gas. Rev.

property of turning syrup of violets green: exerts no sensible action on solution of *pure sugar, albumen, gelatine,* or *milk.* Solid barytes, dissolved in muriatic acid, forms *muriate of barytes,* strongly resembling, in several of its chemical characters, the pure earth. *Physiological action:* Barytes and its compounds, taken into the stomach, produce fatal effects by their influence on the nervous system, and corrosion of the animal texture. The *muriate,* on being injected into the veins, destroys life by coagulating the blood. The *symptoms* of these destructve poisons, and precise nature of the lesions inflicted by them on the human organs, are not known. *Practical application:* Reference to what we have before said of pure barytes, will supersede the necessity of any farther observation here. The *muriate* may be distinguished from other salts; as *not* reddening litmus,* nor turning green syrup of violets; not precipitable by hydro-sulphurets or ammonia; being insoluble in concentrated alcohol; thrown down white by subcarbonates of potash, soda, and ammonia; and affording with sulphuric acid and sulphate of potash, or nitrate of silver, a white precipitate† insoluble in nitric acid. Liquids, suspected of containing this muriate, but not answering to the usual tests, should be treated with aqueous solution of subcarbonate of ammonia: carbonate of barytes will be thrown down; and this, dried and calcined with charcoal, will yield pure barytes. *Sulphuret of barytes,* or the *caustic* earth, may be recovered from *solids* containing this poison, by exposing them, dried and mixed with pulverized charcoal, for two hours, to the action of strong heat. *Treatment:* The *antidotes* of barytes and its compounds are the *soluble sulphates;* which, decomposed by the poisonous substance, yield to it sulphuric acid. Thus, sulphate of barytes is formed, insoluble, and exerting little or no action upon the animal organs. *Solutions of sulphate of soda or sulphate of magnesia* should, therefore, be copiously administered. *Pit-water,* as commonly containing sulphate of lime, may be given, if the former cannot be procured. Vomiting, if not spontaneously excited, should be provoked by the usual means. The after treatment will be regulated by the symptoms. *Lime. Phys. characters* well known.

---

* The translator, in tracing the chemical history of this muriate, erroneously states that it *does* redden litmus, or *tournesol,* as he is pleased to call it. Rev.

† Muriate of silver; nitrate of barytes remaining in the fluid, in the *latter* case.

*Chemical*: exhibits with water, the same phænomenon as barytes. The solution (lime-water) acts, like the former, on syrup of violets, turmeric and litmus; and is affected, in the same way, by carbonic acid and its compounds; but is *not* precipitated by sulphuric acid: yields with oxalic acid, and oxalate of ammonia, a white precipitate; with *Burgundy wine*, a brown, somewhat inclining to violet; with *human bile* (after some hours) a brown; in large quantity with *tea*, an ochre red: disturbs not *albumen, gelatine*, or *milk;* but these mixtures change to green the syrup of violets. *Physiol. action*: destructive, from inflammation of the organs with which it is in contact. *Symptoms and lesions*: The former, those characterizing gastritis and enteritis; the latter, inflammation more or less intense. *Practical application*: The diagnosis will be rendered clear by attention to the chemical history just traced. The presence of lime in matters vomited may be recognized by calcination, which will destroy the animal and vegetable substances in combination, and yield the poison in its caustic state. *Treatment*: that of poisoning by the fixed alkalis (potash and soda.)

*Article* 13. *Species* 13. *Phosphorus. Physical characters* not requiring description. *Chemical*: Combustible on contact with air at the ordinary temperature; giving out smoke and light, and finally disappearing, by combination with oxygen and azote: transformed by nitric acid into phosphoric acid, with extrication of caloric and nitrous gas: insoluble in water, and incapable of decomposing it at the ordinary temperature; soluble in oil (if the temperature be a little raised), and in æther and alcohol; unchanged by tincture of nut-galls; *sugared water, tea, albumen, gelatine, milk*, and *bile*, at the common temperature. *Physiolog. action*: Shortly fatal, if injected, in solution with oil, into the veins: effects variable, according to the state of division; but always destructive, when received into the stomach. Inflammation of the intestinal canal, sometimes inducing sympathetic lesion of the nervous system, seems to be its direct *consequence;* and this depends on the formation of phosphorous, and perhaps phosphoric, acid from combination of the solid phosphorus with the oxygen contained in the stomach and bowels: hence its operation will be more slow, in proportion as the quantity of aliment in the intestinal canal is greater; and air, of course, more completely excluded: but it acts with fearful vehemence and rapidity, when its union with oxygen is favoured by previous mixture in oil; and phosphoric acid is probably the result of this combustion. The *Symptoms and Lesions*, consequent on ingestion of phosphorus, will be modified by circumstances: if the *solid* poison be introduced into a *full* stomach, the symptoms, resembling those of intestinal inflammation, will be slowly developed, and the lesions comparatively slight; but if the combustible have been previously dissolved in oil or æther, pain, vomiting, and nervous commotions, the most severe and terrible, will usher in a speedy death, and the organic lesions will display an aggravated character. *Practical application*: On this we need not pause; since phosphorus can be confounded with no other substance. *Treatment:* Antimonial emetic, if the poison have been swallowed in a *solid form*; if in a state of *division*, large quantities of water, holding *magnesia* in suspension. Thus will be obtained the threefold advantage of arresting the combustion of the phosphorus by expulsion of atmospheric air from the stomach; of exciting that organ to reject its contents; and saturating the phosphorous or phosphoric acid already generated. Intestinal phlegmasia, or nervous affections, supervening, will require prompt employment of the ordinary remedies.

*Article* 14. *Powdered Glass and Enamel.* Many facts are adduced to prove, that glass may be swallowed in considerable quantities with impunity; but this is not invariably the case, as dreadful symptoms have arisen from its introduction into the digestive organs. Portal, in an instance of this kind, recommended an emetic; the stomach having been previously filled with aliment, to protect it from farther injury during the contraction attendant on vomiting; and the noxious substance was happily expelled. The presence of finely powdered glass, in alimentary matters, may be determined by fusion on charcoal, by means of a blow-pipe: for thus a lump of glass will be soon obtained, and the organic substances with which it was mixed, be decomposed by the calcination.

*Article* 15. *Species* 15. *Cantharides.* Physical Characters* well known. *Chemical:* Tincture of cantharides, on mixture with water, assumes a light whitish tinge, and is precipitated white; reddened by litmus, and precipitated of a clear rose colour: becomes canary yellow with prus-

---

* According to Robiquet, cantharides consist of nine distinct substances. For an account of these, and of some experiments made upon the tincture of the *London* Pharmacopœia, we refer the reader to our Miscellaneous Department.—Rev.

siate of potash, and precipitated white slightly inclining to yellow; with subcarbonate of potash, yellow, and precipitated white; with sulphuric and muriatic acid, canary yellow, and precipitated in minute greenish yellow flakes: precipitated yellow by nitric acid (and, after twenty-four hours, an oily reddish matter, resembling in smell fat treated with nitric acid, appears on the surface of the liquid); in clear yellow clots by hydro-sulphurets of potash, soda, and ammonia; and yellowish white, by *tea*. *Physiol. action:* Experiment shews, that the part of the insect which is soluble in oil of almonds,* when thrown into the veins, exerts an influence on the nervous system, and particularly the vertebral column; that the poison introduced into the stomach, produces vehement inflammation of that organ, and sympathetic affection of the nerves, and, if life be some time prolonged, phlegmasia of the internal coat of the bladder; that the destructive effects of its external application on animals, sometimes resemble those which follow its internal employment. *Symptoms:* Six cases are borrowed from various writers, to illustrate the train of phænomena which characterize ingestion, or external use, of this formidable insect. Some of them are detailed with such utter inattention to common decency, that the English translator has very properly suppressed the obnoxious parts, which would but needlessly "insult the delicacy of his readers." The symptoms are, nauseous and disgusting smell; acrid unpleasant taste; copious vomiting, abundant, often bloody, stools, severe epigastric and hypochondriac pains, dreadful colic; burning heat in the bladder, urine sometimes bloody, obstinate and painful priapism; pulse frequent, hard; uncomfortable sensation of heat; intense thirst, sometimes hydrophobia; convulsions, tetanus, delirium. *Organ. Lesions,* resembling those of the other corrosives: sometimes, however, the intestinal mucous membrane presents "fungous tubercles, varices, and ulcerations; and the internal membrane of the bladder and genital organs, vestiges of inflammation." *Practical application:* Animal substances display, with various menstrua, very complicated phænomena; and the results of chemical tests are little to be depended on. In such researches, the physical properties of the poison constitute the most solid criterion; and to these should be united investigation of the

* Alcohol, injected into the jugular vein of a dog, produced the same, almost instantaneously, fatal effect as tincture of cantharides.

history, symptoms, and lesions, offered by the individual case. *Treatment:* That generally employed in poisoning by the other corrosives; mild emetics of oil: when tetanus supervenes, frictions with a liniment of oil, laudanum, and ammonia; opium and musk internally.

*Chapter* II. *Class* II. *Of Astringent Poisons.* So called, from frequently producing contraction of the large intestines, particularly the colon. Inflammation of the intestinal membranes, and action upon the nervous system, may also frequently result. This class includes only preparations of lead.

*Article* 1. *Species* 1. *Lead.* Varieties here enumerated eight. From these we shall select *Acetate of Lead.** *Physical characters* requiring no description. *Chemical:* Fusing and finally yielding metallic lead, mixed with yellow protoxyde, and an acid product of fœtid smell, on being exposed to heat in a crucible. The quantity of metal will be greater, if the acetate, previously combined with charcoal, be long submitted to the action of strong heat: decomposed with effervescence by sulphuric acid, and giving out vapours of acetic acid. Soluble in distilled water. The solution, limpid, transparent, colourless, is precipitated white by sulphuric acid and sulphates of potash, soda, and ammonia; by subcarbonate of soda (one of its most delicate tests); by ammonia; by muriatic acid and the muriates; and by spring water:† black by sulphuretted hydrogen, gaseous or dissolved in water, and hydro-sulphurets; canary yellow by chromic acid and chromate of potash; yellowish white by tincture of nutgalls and *tea;* white by *albumen;* in white flakes, assuming, when dry, the colour and consistence of glue, by *broth: Gelatine* does not sensibly affect it: *Burgundy wine* forms with it insoluble tartrate of lead; *human bile,* a precipitate composed of protoxyde and animal matter: in large quantity, it coagulates *milk;* added in the proportion of one part to fifty of milk, it does not disturb the animal fluid; and hydro-sulphurets throw down from the mixture a grey precipitate inclining to black. All the precipitates yield to the action of heat *metallic lead. Physiol. action:* Researches on the bodies of individuals destroyed by *saturnine emanations,* have led Merat to the inference, that they "exert their deleterious influence upon the *muscular*

---

* *Super-acetate* of the London Pharmacopœia.—Rev.

† In consequence of the sulphates and carbonates which spring water holds in solution.

coat of the intestinal tube, and especially on the nerves distributed to these muscles:" and he thinks that such opinion derives support from "the constriction which prevails in certain portions of the intestine; a property inherent in the muscles, and not possessed by other systems. If the lead exercised its influence on the *mucous* membrane, there would be a more copious secretion of the fluid peculiar to this structure; and a kind of dysentery or diarrhœa would ensue; whereas, in reality, there is constipation. Still less does this metal exert its action on the *peritoneal* covering of the intestines; for, in that case, we should have a species of peritonitis signalized by fever, abdominal tension, inflation, and heat. But none of these phænomena exist. On the contrary, we find flattening of the abdomen, insensibility to pressure, asphyxia." Injected into the veins, the acetate of lead may destroy life, apparently by its action on the nervous system; although it operates less powerfully than most of the other metallic salts. Lastly, when introduced into the stomach in large doses, this compound destroys life, even although vomiting be induced. "Corrosion of the intestinal canal" is unquestionably the result of its ingestion in a *solid* state; destructive influence upon the nervous system, in the *liquid* state, and when time for the passage of the poison into the absorbents has been allowed. *Symptoms:* These are elucidated by the narration of six interesting cases. The principal are, colic, at first of short duration, soon recurring, and eventually becoming constant; alvine discharges painful and difficult, the excrement generally yellow, hard and round, like the dung of some quadrupeds, but in the progress of the disease growing soft and almost watery; nausea and vomiting, sometimes continuing several days, but commonly ceasing after the second day of the treatment,—the liquid ejected, greenish or blackish, and bitter; (the vomiting principally takes place when the pains are very acute); retraction of the abdomen, and sinking in of the navel towards the spine; anorexia, sleeplessness, and anxiety, sometimes very great; no fever, however violent be the pain; urine natural. The invasion of these symptoms may be rapid or slow: they are, in general, successively developed; but, in some rare cases, all at once. Head-ache, or borborygmus, seldom occurs. In two instances, M. Orfila has observed frequent eructations producing in the mouth the sensation of a saccharine body. Constipation commonly exists; but there is sometimes tormenting diarrhœa. The pain of the umbilical region is, for the most part, signally relieved by compression; but there are cases in which the slightest pressure is intolerable. Other common or occasional symptoms are, countenance pale or yellowish, and shrunk during the pain; respiration disordered; limbs painful; delirium; jaundice; retraction of the testicles; convulsions. The reader will bear in mind, that this description applies principally to the disease induced by exposure to the influence of saturnine *emanations;* or, in other words, the painter's colic. The phænomena, resulting from *ingestion* of a saturnine poison, are, according to Plenck, dryness of the mouth; sense of strangulation, vertigo, cough, asthma, hiccough; chronic inflammation of the abdominal viscera; ischury, dysury, loss of voice, cold sweats, death. *Organic Lesions:* When the poison has been swallowed in large doses, inflammation of the free surface of the gastric mucous membrane, sometimes extending to the adherent, or that surface in contact with the muscular, and to the other membranes: sometimes, in the interior of the organ, black points or spots, of various extent, dependent on extravasation of venous blood, or injection of the vessels with it: lastly, the interior of the stomach and whole intestinal tube of animals, which have *not* vomited after receiving a large dose of solution of acetate of lead, has exhibited a thickish ash-coloured membranous lining, readily separable, and probably arising from decomposition of the salt by the mucous, bilious, and other gastric fluids. The subjacent mucous membrane was of a deep grey colour throughout its substance, and seemed to have exerted a similar action upon the poison. In fatal cases of disease from saturnine *emanations,* no morbid change is observed, except contraction of the colon; nor can any trace of the metal be detected in the intestinal canal. Its influence seems to be directly exerted upon the nervous system.—*Practical Application.* 1. *The patient living: the remnant of the poison may be procured.* The poison, uncombined, is distinguishable by the facility with which it resumes the metallic form on calcination with charcoal; and by the action of the wonted tests, particularly sulphuric, chromic, and muriatic acids, alkalis, hydro-sulphurets, and subcarbonate of soda. Adulteration of *wine* by litharge, may be detected by distilling part of it in a retort to obtain the alcohol, and calcining the residue with charcoal; and, secondly, by trying the other portion by the usual re-agents.* The impregnation of *beer*, from having been fermented in leaden vessels, will be determined by a similar process: admixture of *cerusse* with *bread*,† by calcining the bread or flour so as to reproduce metallic lead; and, again, by pouring upon another portion, acetic acid at the ordinary temperature: thus, acetate of lead, answering to the usual tests, will be formed. Chemistry can throw no light upon poisoning by saturnine *emanations*. Neither the urinary nor fæcal excrements of patients, suffering from them, have yielded a trace of the metal to the most rigid analysis. In such cases, the history and existing symptoms of the disease can form the only ground of diagnosis.—II. *All the poison swallowed: the matter vomited, and that found in the intestinal canal after death, may be examined.* Separation of the *fluid* from the *solid* portion of this matter, trial of the former, and of the distilled water in which the latter has been boiled, by the ordinary re-agents; and calcination of the solid portion with potash and charcoal; as well, if necessary, as of the "mucoso-flocculent layer" lining the interior of the intestines, and of such parts of their mucous membrane as may display alteration; are the measures indicated under this combination of circumstances. *Treatment:* Navier has extolled *alkaline sulphurets* as the antidotes of saturnine poisons. But the experiments of M. Orfila shew, that animals, to which acetate of lead has been given, and afterwards decomposed in the stomach, perish as though the supposed antidote had not been administered. This fact is afterwards explained: the *sulphuret of potash* is, itself, when taken in large doses, a powerful corrosive poison. The sulphates of magnesia, potash, and soda, possess the property of quickly decomposing saturnine preparations, and forming with them an insoluble and inert sulphate of lead. *Water, largely given, and containing, in each two pints, three or four drachms of sulphate of magnesia, or other soluble sulphate, is, therefore, the real antidote to the acetate, and other active preparations, of lead.* We cannot allow ourselves to detail the methodic treatment‡ which has

* Here no attention should be paid to those precipitates which present varieties of colour, dependent on the action of the wine.

† An adulteration frequently practised by bakers, to render the bread more heavy, and whiter.

‡ The translator, in describing this treatment, has been guilty of one capital omission, and three important misconstructions. We would advise him to collate pages 486-7 of his work carefully with the original.—Rev.

been long and successfully employed against the painter's colic in the Parisian hospital *La Charité:* it consists principally of emetics, purgatives, sassafras, guaiacum, opium, and anodyne injections. Merat has cured the disease by tartrite of antimony alone, largely diluted with water, and administered both in drinks and injections.

*An Appendix*, containing three papers, terminates the volume. I. *Upon Iodine.* This newly discovered substance, shews itself under the form of small plates, of metallic lustre, bluish colour; tenacity inconsiderable; resembling, in appearance, carburet of iron; smell like that of sulphurane.* Specific gravity 4,946. It is little soluble in water, and communicates to it a slight amber-yellow tinge; has a strong affinity for hydrogen; and decomposes almost all organized bodies by combining with this principle. Its precise action on animal and vegetable substances is yet unknown. *Physolog. action:* Introduced into the stomach in small doses, it excites vomiting; destroys life, in the quantity of a drachm, if the œsophagus be tied; and in the dose of two or three drachms, even if the ligature have not been made, and some hours elapse ere vomiting ensue. It acts by producing ulceration of the mucous membrane; and is, in fact, *a corrosive poison.* No fatal consequences result from its *external* application.

II. *On the Antidotes of Arsenic and corrosive Sublimate.* Many experiments are here recorded to disprove the assertion made by Dr. Berrand that *charcoal* is an antidote of these poisons.

III. *On Hydrogenized Sulphuret of Potash,* (sulphuret of potass dissolved in water). M. Orfila here corroborates a suspicion, excited during his experiments on the antidotic properties of this substance, that it may justly be regarded as *one of the most powerful corrosive poisons.* We cannot do better than transcribe, in his own language, the inferences drawn from several experiments on this subject.

"Hydrogenized sulphuret of potash, introduced into the stomach, occasions death by its action on the nervous system, and corrosion of the gastric membranes: This corrosion is more slight in proportion as the dose of the sulphuret administered is *larger;*

---

* Sulphurane, a compound of sulphur and chlorine, having the smell of *sea weed.* Had the translator been conversant, as he ought to have been, with his dispensatory, he would not have blundered at the French term, *soufre oxi-muriate,* and confounded the English reader with the barbarism of a *literal* construction. REV.

because then the nervous phænomena are much more intense." Lastly, this substance "thrown into the torrent of circulation, destroys life by acting particularly upon the nervous system."

We need not delineate the *Physical characters* of sulphuret of potash. *Chemical:* It decomposes water partially, and passes into the state of hydrogenized sulphuret, soluble in the undecomposed portion of the fluid. The solution, of a yellow or red colour, is instantly decomposed by the strong acids, sulphuretted hydrogen gas being disengaged and sulphur thrown down: precipitated orange-yellow or red-brown by tartrite of antimony and *other soluble* antimonial preparations; white, becoming yellowish on a fresh addition of the sulphuret, by arsenious acid: With corrosive sublimate, acetate of lead, acid nitrate of bismuth, and the salts of copper, it yields a black precipitate.

To pass any laboured eulogium upon the first volume of M. Orfila, after the analysis we have just concluded, and the reception which it has experienced, not only in this island, but from all the more enlightened nations of the Continent, would be superfluous. That it is not unexceptionable in style and arrangement, and offers occasional examples of hasty and illegitimate induction, we are well aware. But admitting all these blemishes to the fullest extent, and with the work of the learned Gmelin* before us, we hesitate not to declare it by far the most perfect system of doctrine upon mineral poisons that has yet appeared; and the noblest tribute which the union of chemistry and anatomical research, has ever offered to practical medicine.

Equally needless is it to pronounce an unfavourable sentence upon the labours of the English Translator. Elegance of style, in works of this nature, should, we willingly allow, be never, for a moment, put in competition with the more essential objects of a scientific book, clearness and fidelity. But wantonly to violate the correctness of such a writer as M. Orfila, and upon such a subject, constitutes an offence which all the rigours of our critical purgatory would in vain be inflicted to expiate.† We

---

* *Allgemeine Geschichte der thierischen und mineralischen Gifte. Von Johann Frederich Gmellin.* Rev.

† If, after the numerous errors which we have noticed, any evidence were wanting that these sheets went to press without revisal or corection, the *introduction* affords a proof of it, curious as conclusive. Orfila, in designating the six classes of poisons which form the basis of his arrangehave scrutinized with no malignant eye, the pages of the translation to swell the catalogue of blunders, and exult in its exposure. Those only, which are conspicuous from their situation or magnitude have we paused to notice. Enough yet remain in their lurking holes to form a legion of *corrigenda.* All imputation of prejudice and personal feeling will be most effectually averted by the declaration that, at the moment of penning this sentence, we are utterly ignorant of the name and situation of the gentleman, whose labours are the object of our just criticism: although, if we mistake not, in *another more recently published and anonymous "translation from the French,"* traces of the same rapid and negligent hand are clearly perceptible. Whoever that gentleman may be, we trust that, in the execution of the remaining volume, he will profit by admonitions, which, though delivered in the rugged intrepidity of conscious truth, are calculated rather to excite gratitude than resentment, if he possess a mind generous enough to appreciate aright the untainted purity of their source.

For the sake of including both the original and translation of M. Orfila's work under one review, we have preferred delay to the ostentation of an earlier, and the disadvantages of a less useful and comprehensive, notice. The second volume of the former has long been before us. We shall proceed in our analysis, as soon as the English Translator has favoured us with the conclusion of his labours.

ment, commits a mistake; giving to two of them, titles nearly synonymous. The translator, aware of some inaccuracy, pauses not to investigate; but manfully dashes through the difficulty by naming only *five* of the *six* classes just previously announced. Now had he only explored a few pages farther, the precise nature of the error, and mode of rectification, would have been at once obvious. REV.

