# Errata

**Published:** 1816-08

**Authors:** 


### ERRATA.

P. 48, l. 15, *for* asphyxia, *read* apyrexia.

